# Managing Asthma Well and Sustainably – Patient Perspectives Explored

**DOI:** 10.1111/hex.70751

**Published:** 2026-07-03

**Authors:** Imogen Le Couteur, Sean Docking, Katy Bell, Alexandra Barratt, Rebecca Haddock, Darlene Cox, Kate Wylie, Luise Kazda

**Affiliations:** ^1^ Wiser Healthcare Research Collaboration, Sydney School of Public Health, Faculty of Medicine and Health The University of Sydney Sydney NSW Australia; ^2^ Musculoskeletal Health and Wiser Health Care Units, School of Public Health and Preventive Medicine Monash University, Melbourne VIC Australia; ^3^ Asthma Australia Sydney NSW Australia; ^4^ HealthCare Consumers' Association Canberra ACT Australia; ^5^ Doctors for the Environment Australia Melbourne VIC Australia; ^6^ HEAL Global Research Centre University of Canberra Canberra ACT Australia

**Keywords:** asthma, evidence‐based medicine, general practice, patient perspectives, qualitative methods, respiratory inhalers, shared decision making

## Abstract

**Context:**

Asthma affects 300 million people worldwide. The widespread use of short‐acting beta_2_‐agonist (SABA) relievers, commonly delivered via pressurised metered dose inhalers (pMDIs), is contrary to evidence‐based guidelines. SABA overuse is associated with poor asthma control and high carbon emissions. Reducing reliance presents a dual opportunity to improve patient outcomes while decreasing environmental impact, yet patient perspectives remain unclear.

**Objective:**

The aim of this study was to explore the perspectives, understanding, and preferences of a sample of Australians living with asthma regarding inhaler types, medication options, self‐management, and environmental impact.

**Methods:**

We conducted in‐depth interviews with adults who regularly used inhalers to manage their asthma (*n* = 23), recruited via consumer health networks. Participants completed an online pre‐interview survey on demographics, asthma history and inhaler use. Interviews were semi‐structured, following a guide developed with advice from the consumer panel. Data were analysed thematically using an inductive framework. Survey data were summarised descriptively.

**Results:**

Predominantly female (91%) participants had limited awareness of different device types and their environmental impact, with many surprised by pMDI emissions. While most valued environmental sustainability, they prioritised access to fast‐acting SABA pMDIs, perceived to be the most effective emergency treatment by many. Familiarity, psychological comfort, and concern about reliable relief during acute asthma attacks were key barriers to switching device types. Health professional recommendation strongly influenced choice of inhaler, with most participants relying on clinician advice and expressing limited involvement in decision‐making. Barriers to accessing care and information, such as cost, time and rurality, contributed to knowledge gaps and potential overreliance on SABAs. Positive experiences with asthma care nurses and pharmacists highlighted opportunities for multi‐disciplinary care teams to improve support.

**Conclusion:**

Environmental concern is important to patients but, alone, is insufficient to drive change in asthma management. Treatment is shaped by familiarity, habit and fear of exacerbations, with strong reliance on clinician expertise.

**Patient and Public Contributions:**

A consumer panel of asthma patients provided lived‐experience insights during study conception, helped shape interview materials, and reviewed our findings and conclusions. Asthma Australia (Australia's peak asthma consumer advocacy body) and the Health Care Consumers' Association were engaged in this project since inception of the study idea, collaborated in co‐designing the funding proposal, recruiting participants and their representatives contributed to the research as co‐authors. Their involvement ensures our research reflects real‐world experiences and perspectives.

## 1 Introduction

Asthma is a chronic respiratory condition affecting approximately 300 million people globally [1], including 2.8 million Australians (~11% of the population), with higher prevalence in adult women than men (13.8% vs 9.2%) [2]. Asthma is characterised by airway inflammation and hyperresponsiveness, with episodes of acute airflow obstruction that cause cough, wheeze, and shortness of breath [1]. Treatment typically involves a combination of preventer and emergency reliever medication delivered via one or more respiratory inhalers.

In Australia, around 25 million inhalers are purchased anually [3]. Of these, 15 million are dispensed by prescription (~1/3 as relievers; ~2/3 as preventers or combination inhalers), of which around 60% are pressurised Metered Dose Inhalers (pMDIs) and the remainder largely dry powder inhalers (DPIs) [4]. An additional, estimated 10 million short‐acting beta_2_ agonist (SABA) relievers are sold over‐the‐counter, without prescription or prior medical consultation [3]. This happens because, in Australia, SABA inhalers are classified as Schedule 3 (pharmacist‐only) medicines, allowing non‐prescription supply from pharmacies. While this SABA exemption from prescribing ensures universal and equitable access to these relievers, it limits the ability to monitor their usage [3]. Notably, 98% of all SABA relievers sold in Australia are pMDIs [4].

93% of Australian adults with asthma report using SABA inhalers for symptom relief [5]. However, these are frequently overused (3 or more inhalers per year), indicating suboptimal asthma control and increased risk of severe exacerbations including emergency department visits and hospitalisations [6]. Recently updated national and international clinical guidelines no longer recommend SABA‐only treatment for adults and adolescents [7, 8]. Instead, guidelines recommend inhaled corticosteroid (ICS) plus long‐acting beta_2_‐agonist (LABA) with rapid onset (formoterol) combination inhalers as first‐line treatment. In Australia, these are available as low‐dose, as‐needed anti‐inflammatory reliever (AIR‐only) treatment for those with mild, intermittent symptoms, or as regular maintenance‐and‐reliever therapy (MART) in patients with more frequent or severe symptoms, or at high risk of exacerbation [9]. Both are readily available as pMDIs or DPIs.

Beyond their clinical limitations, SABA pMDIs contain hydrofluorocarbon propellants. These are potent greenhouse gases that are up to 3,200 times more climate damaging than carbon dioxide (CO_2_) over 100 years [10], meaning that pMDIs produce between 20 and 30 times as much CO2e emissions as DPIs [11]. They contribute approximately 450,000 tonnes of CO_2_ equivalent annually [4], around 3% of Australia's healthcare services carbon footprint [12], which is comparable to the emissions emitted from over 100,000 petrol‐powered cars driven for 1 year [13]. SABA pMDIs account for an estimated 80% of these emissions, with the remainder from preventer or combination pMDIs [4]. In the UK, poorly controlled asthma, mainly due to SABA overuse, generates an eightfold higher excess carbon footprint per person than well‐controlled asthma [14]. Thus, reducing SABA over‐reliance and supporting evidence‐based asthma management presents a dual opportunity to optimise patient outcomes while reducing environmental impact through decreased need for high‐emissions relievers and healthcare use [15].

While recent international studies including surveys and mixed‐methods research conducted in the UK, New Zealand and Canada suggest that provider and patient awareness of environmental impact of pMDI inhalers is low, they also indicate that patients are often open to considering more environmentally sustainable inhalers when informed [16, 17, 18, 19]. In Australia, however, awareness of the environmental impact of inhalers remains low among patients, as this is an emerging topic largely confined to policy, academic and clinical discourse with information mainly disseminated through these channels (e.g. clinician‑focused National Asthma Council guidance [20] or policy documents such as the National Health and Climate Strategy [21]), with very limited translation into patient‑facing resources (e.g. by Asthma Australia [22]). To the best of our knowledge, no research has explored Australian consumers' awareness or the role of environmental considerations in inhaler decision‐making. There is also a critical evidence gap regarding how patient perspectives and preferences support or impede a transition to high‐quality, low‐carbon asthma care.

This study aims to investigate the perspectives, understanding and preferences of Australians living with asthma regarding their management. Specifically, we sought to elicit patients' knowledge, awareness, experience and attitudes towards 1) different inhaler types (e.g. pMDIs vs DPIs); 2) medication options (e.g. guideline‐recommended ICS‐containing vs SABA‐only/SABA over‐reliance); 3) their own asthma management; and 4) the environmental impact of inhalers. We also explored perceived barriers and facilitators to optimised asthma care in practice. By directly exploring patient perspectives, we intend to generate evidence to inform interventions that empower patients to engage in informed, shared decision‐making with healthcare professionals, towards optimised asthma management and environmentally sustainable care.

## 2 Methods

### 2.1 Design

This study was conducted within an interpretivist paradigm, which assumes that reality is socially constructed and that knowledge is generated through understanding individuals' experiences and meanings [23]. The study used in‐depth, semi‐structured interviews to address the study objectives. Methods are reported in accordance with the Consolidated Criteria for Reporting Qualitative Research (COREQ) [24] (Supplement 1). The research team has no financial or commercial interests related to the study topic and the study was conducted independently of any industry involvement.

### 2.2 Participants and Recruitment

Participants were eligible if they met the following criteria: (1) self‐reported diagnosis of asthma; (2) aged 18 years or over; (3) a current (within the last 6 months) and regular (at least once a month) user of one or more types of respiratory inhalers; and (4) currently residing in Australia. Participants were invited to participate in an interview through a social media post (Facebook) in November 2024 by the Australian Health Care Consumers' Association, and e‐newsletter advertisements through Asthma Australia (Australia's peak asthma consumer body) in December 2024. Potential participants followed a link to the study's online participant information statement, online consent form and pre‐interview survey. All eligible individuals were contacted to organise a time for the interview.

### 2.3 Data Collection

All participants first completed a brief pre‐interview survey deployed through online platform Qualtrics to ascertain eligibility and collect participants' basic demographic data, health literacy, time since initial diagnosis, self‐reported symptom severity (mild, moderate, severe) and disease control information as well as inhaler use history (Supplement 2). Disease control was established through the 5‐item Asthma Control Questionnaire‐5 (ACQ5), a validated and standardised tool to assess self‐reported asthma symptom control [25] with the final score ranging from 0 to 5 (< 0.75 = well‐controlled, indicating effective current treatment, no limitations on daily activities; 0.75 to 1.5 = partially controlled, treatment adjustment could be considered, daily activities somewhat impacted by symptoms; > 1.5 = not well‐controlled, poor control, higher risk of exacerbations, significant limitations on daily activities) [26]. Health literacy was measured using a single item screening tool [27].

The interview guide (Supplement 3) was co‐developed by the research team with clinical and consumer input and piloted with one individual with asthma, to ensure clarity and accessibility of questions. Minor modifications were made to the interview guide following initial interviews to ensure study aims were effectively investigated. The interview guide commenced with exploring participants' views, knowledge and experiences with reliever and preventer (including AIR‐only and MART) inhalers as well as their general asthma management experiences. Next, participants' awareness and views on SABA over‐reliance and associated risks were explored using a prompting statement ‘*People commonly rely on Ventolin/SABA/blue puffers [depending on participant use of language] for quick relief. But using this type of inhaler more than occasionally isn't generally recommended. Have you ever heard this before?*’ The statement was intentionally kept quite general and unspecific to avoid leading and biasing responses. Finally, supported by a visual prompt (Supplement 4), perspectives and awareness of inhalers' environmental impact was also investigated. One‐on‐one interviews were conducted via videocall or telephone with one researcher, trained and experienced in qualitative research methods. Interviews were audio recorded and manually transcribed verbatim (IL). Participants were compensated for their time with an online gift card. Interviews were conducted until thematic saturation was reached, defined as the point at which no new themes emerged from the data and agreed upon by two researchers (IL, LK).

### 2.4 Data Analysis

Data were analysed thematically using an inductive framework approach [28]. One investigator reviewed the transcripts and developed initial impressions (IL); a quarter of transcripts were independently reviewed by another author (LK). Initial codes were iteratively developed and discussed and informed the coding framework. All transcripts were then coded and analysed thematically in NVivo 15 [29] (IL). Salient themes were discussed and interpreted amongst members of the core research team. We used descriptive analyses to summarise quantitative data from the online survey in Microsoft Excel [30].

## 3 Results

Thirty adults living with asthma provided consent to participate in the study and (at least partially) completed the pre‐interview survey. All of these were invited to participate in an interview via email. Four candidates declined due to timing issues and three did not respond. Interviews were conducted with the remaining 23 participants between November 2024 and January 2025. Average interview length was 37 min (range: 25–54 min), average transcript length was 14 pages (range: 8–24 pages). Participant demographic and clinical characteristics are described in Table 1. The mean interview participant age was 50 years (SD = 11.7; range: 34–75 years), and the majority were female (91%, *n* = 21). Most lived in metropolitan areas (74%, *n* = 17), and almost half in Victoria (43%, *n* = 10). The majority held at least a bachelor's degree (83%, *n* = 19) and had high health literacy (91%, n = 21).

**Table 1:** Participant characteristics.

	Completed survey (n = 30)		Completed survey and interview (*n* = 23)	
	*N*	%	*N*	%
*Sociodemographic characteristics*				
Age range				
< 30	2	7%	0	0%
30–39	4	13%	3	13%
40–49	14	47%	13	57%
50–59	2	7%	1	4%
60–69	6	20%	4	17%
> 69	2	7%	2	9%
Gender				
Female	27	90%	21	91%
Male	3	10%	2	9%
State				
ACT	5	17%	5	22%
NSW	4	13%	3	13%
NT	0	0%	0	0%
QLD	1	3%	1	4%
SA	3	10%	3	13%
TAS	2	7%	1	4%
VIC	15	50%	10	43%
WA	0	0%	0	0%
Area				
Metropolitan	23	77%	17	74%
Regional or remote	7	23%	6	26%
Main language spoken at home is English				
Yes	28	93%	21	91%
No	2	7%	2	9%
Highest level of education				
Graduate or postgraduate degree	11	37%	9	39%
Undergraduate degree or certificate III/IV	17	57%	13	57%
High school	2	7%	1	4%
Confidence in filling out medical forms alone				
Extremely/quite confident	28	93%	21	91%
Somewhat confident	2	7%	2	9%
Not at all/a little confident	0	0%	0	0%
*Diagnosis and severity*				
Time since asthma diagnosis				
Less than 6 years ago	3	10%	0	0%
6 to 10 years ago	3	10%	3	13%
More than 10 years ago	24	80%	20	87%
ACQ5 scores^a^ stratified by self‐rated asthma severity				
Mild	12	40%	11	48%
Well‐controlled asthma (< 0.75)	3	25%	3	27%
Partially controlled asthma (0.75–1.5)	7	58%	7	64%
Not well‐controlled asthma (> 1.5)	2	17%	1	9%
Moderate	16	53%	10	43%
Well‐controlled asthma (< 0.75)	1	6%	1	10%
Partially controlled asthma (0.75–1.5)	1	6%	1	10%
Not well‐controlled asthma (> 1.5)	14	88%	8	80%
Severe	2	7%	2	9%
Not well‐controlled asthma (> 1.5)	2	100%	2	100%
*Inhaler use*				
Length of time using inhalers				
Less than 6 years	3	10%	0	0%
6 to 10 years	4	13%	4	17%
More than 10 years	23	77%	19	83%
Median number of inhalers used per participant (IQR)	2 (1)	—	2 (1)	—
Current type of inhaler device(s) used^b^,^c^,^d^				
DPI	11	38%	10	43%
pMDI	25	86%	20	87%
SMI	4	14%	3	13%
Current inhaler medication(s) used^b^,^c^,^d^				
ICS/Formoterol (AIR‐only or MART)	9	31%	9	39%
ICS/LABA	8	28%	7	30%
ICS/LAMA/LABA	4	14%	3	13%
ICS	3	10%	1	4%
LAMA	4	14%	3	13%
SAMA	3	10%	2	9%
SABA	22	76%	19	83%
SABA‐only	4	14%	2	9%
Frequency of inhaler use				
Daily	22	73%	16	70%
2‐6 times per week	2	7%	2	9%
Once a week	2	7%	1	4%
Occasionally	4	13%	4	17%
Has asthma management plan				
Yes	16	53%	12	52%
No	14	47%	11	48%

Abbreviations: ACQ5, Asthma Control Questionnaire; AIR, Anti‐inflammatory reliever; DPI, Dry Powder Inhaler; ICS, Inhaled corticosteroids; LABA, Long‐acting beta2‐agonist; LAMA, Long‐acting muscarinic antagonists; MART, Maintenance and reliever therapy; pMDI, Pressurised Metered Dose Inhaler; SABA, Short‐acting beta2‐agonist; SAMA, Short‐acting muscarinic antagonists; SMI, Soft Mist Inhaler.

^a^ Overall score obtained from average of ACQ5 survey questions: (1) On average, in the last week, how often were you woken by your asthma during the night?; (2) On average, in the last week, how were your asthma symptoms when you woke up in the morning? (3) In general, in the last week, how limited were you in your day‐to‐day activities because of your asthma?; (4) In general, in the last week, how much shortness of breath did you experience because of your asthma?; (5) In general, in the last week, how often did you wheeze?

^b^ Some participants reported using more than one inhaler.

^c^ As reported in online survey and in interviews.

^d^ *n* = 29 for survey only participants, one participant dis not answer these questions.

Participants had predominantly been diagnosed over ten years ago (87%, *n* = 20), and almost all participants rated their asthma as mild (48%, *n* = 11) or moderate (44%, *n* = 10) within the last month. ACQ5 scores indicated that only three of eleven (27%) with self‐reported mild asthma, and one of ten (10%) with self‐reported moderate asthma, had well‐controlled symptoms. Overall, eight (35%) participants' asthma was partially controlled, and in almost half (*n* = 11, 48%) it was not well‐controlled.

The median number of inhalers used was 2 (interquartile range = 1). Most participants (87%, *n* = 20) were currently using pMDIs, and around half were using DPIs (43%, *n* = 10). The most common inhaled medications used were SABA (83%, *n* = 19) (2 participants indicated SABA‐only, the others in combination with preventer medication), followed by ICS‐formoterol (39%, *n* = 9) or ICS‐LABA (other than formoterol) inhalers (30%, *n* = 7). Most participants used their inhalers daily (70%, *n* = 16), and just over half currently had an asthma action plan (an individualised written plan to help a patient recognise worsening symptoms and provide clear response instructions [31]) in place (52%, *n* = 12).

We identified five main themes from the interview data: (1) Limited awareness of inhaler device types and their environmental impact; (2) Perceived tension between environmental values and asthma care; (3) Need for systems change and limited patient responsibility; (4) Influence of fear, familiarity and ease of use on preferences and willingness to change; (5) The role of health professionals, access to care and information. Illustrative quotes are provided, along with participant's ID, gender, age, symptom severity and, where relevant to the context, type of inhaler and medication used.

### 3.1 Limited Awareness of Inhaler Device Types and Their Environmental Impact

Many participants had limited or vague understanding of the device type of inhaler they were using, often confusing pMDIs and DPIs. While around half could identify their inhaler type when presented with images and information, most were unsure about the differences between devices.

I'm assuming that the Ventolin [brand name for SABA] is the pressurised one. My preventer is the dry dose one. I don't know what the actual difference between them is or why one is one and not the other. Really no idea.

(P21, female, 37, partially controlled)

Several participants were unfamiliar with DPIs altogether, they had *‘never, ever seen’* (P2, female, 62, partially controlled) or *‘never heard of’* (P12, female, 35, not well‐controlled) DPIs. pMDIs, on the other hand, particularly the Ventolin brand (SABA), were often described as the default or *‘normal inhaler*…*that's all I know it as, as the standard inhaler’* (P12, female, 35, not well‐controlled).

Those who knew differences between device types focused on physical characteristics:

Well, they work differently. And they taste differently…these ones [pMDIs], you don't have to breathe in…you have to breathe in just normally, whereas the others [DPIs] you have to pull it in. The other ones you have to kind of click something to, you know, activate whatever is coming out. Just push the button.

(P9, female, 48, partially controlled)

Awareness of the environmental impact of inhalers was generally low. Two participants who were aware, had earlier identified emissions impact as a limitation of pMDI inhalers.

I do a bit of work in the health sector, and people do talk about the propellent ones being not so good for the environment.

(P1, female, 45, partially controlled, DPI)

However, most participants were surprised to learn about the emissions difference between inhaler types presented during interviews (Supplement 4) and none were familiar with the extent of pMDI emissions or had ever discussed the environmental impact of inhalers with a healthcare provider.

Wow. I had no idea about that…I'm really into saving the planet…but I had never, ever heard that about the inhaler. That is just amazing, like you've blown my mind.

(P4, female, 73, not well‐controlled, pMDI)

I was actually really shocked about that. I had no idea.

(P10, female, 48, not well‐controlled, pMDI)

I just never thought about using medicine as against the environment.

(P17, female, 45, not well‐controlled, DPI and pMDI)

Many participants felt that few people with asthma would be aware of this information, and young people in particular would be concerned by the impact,

I think especially young people should and would be thinking about this if they were aware, maybe most people wouldn't be aware.

(P16, female, 34, partially controlled, pMDI)

One participant rejected the idea of environmental impact being relevant in clinical decision‐making:

So, they're going to start controlling us now, with what medicines we use because it's bad for the environment…it's life‐saving medication. We need it. I feel like are they trying to take it away from us?.

(P17, female, 45, not well‐controlled, DPI and pMDI user)

Some others expressed concerns that pMDIs could be *‘phased out given the emissions’* (P13, female, 41, well‐controlled, DPI and pMDI).

Many participants showed environmental concern in spontaneously raising issues of waste and recyclability of inhalers.

You can't recycle them as far as I'm aware. So even though the dry powder inhaler is definitely 100% better environmentally, I wish we could take it that step further.

(P19, female, 52, not well‐controlled, DPI and pMDI)

These concerns around plastic pollution seemed more tangible and, thus, dominated sustainability discussions with some participants as well as minimising the perceived differences in environmental impacts between inhaler types:

And you just look at it, and you're looking at plastic. […] Yeah, it's probably much the same, you know, comparatively. Yeah. So that's a downfall of both of them.

(P19, female, 52, not well‐controlled, DPI and pMDI)

### 3.2 Perceived Tension between Environmental Values and Asthma Care

Almost all participants expressed a desire to *‘try to do what's good for the environment’* (P18, female, 62, well‐controlled), identifying environmental sustainability as an important personal value. However, many participants perceived a tension between their environmental values and their need for lifesaving asthma care – describing this as a ‘*fine line between what's good for the environment and people's lives’* (P22, female, 67, not well‐controlled) ‐ and prioritising their need for a *‘safe and effective’* (P19, female, 52, not well‐controlled) trustworthy inhaler:

As much as it saddens me that it's causing so much emissions. I suppose I have to balance out what I need.

(P18, female, 62, well‐controlled, pMDI SABA‐only)

Many participants expressed that asthma was *‘too dangerous [to] muck around with’* (P22, female, 67, not well‐controlled) and ultimately held a firm belief that healthcare needed to be prioritised over environmental concerns,

X‐rays aren't supposed to be very good for the environment. But we need them…And if you have an asthma attack and you take a metered dose inhaler, you need it.

(P22, female, 67, not well‐controlled, DPI SABA, pMDI ICS‐formoterol and LABA)

SABA pMDIs especially, were frequently described as a ‘*lifeline’* (P22, female, 67, not well‐controlled) and ‘*lifesaving’* (P3, female, 42, partially controlled). Even those participants with well‐controlled asthma due to regular preventer use who rarely needed their SABA pMDI valued it for reassurance:

I mean the Ventolin's just really good to have and I love how instant it is.

(P8, male, 41, well‐controlled, DPI ICS‐LABA and occasional pMDI SABA)

### 3.3 Need for Systems Change and Limited Patient Responsibility

Some participants felt that *‘the patients don't really get a choice’* (P20, female, 44, not well‐controlled) around medication or device type decisions. Others also questioned whether the emissions from their personal inhaler use were significant, not by denying their impact, but by positioning inhaler use as essential to health and survival, and therefore less suitable to change than discretionary, non‐essential sources of emissions, limiting their perceived responsibility:

People who get the coffee cups everyday, people who fly all the time, like they're the people that can make the biggest change quickly, compared to people who are actually on lifesaving medications… Me personally as one little person, I'm not gonna be too worried.

(P3, female, 42, partially controlled asthma, pMDI ICS‐formoterol and SABA)

I just feel as though I'm no good to the world if I can't breathe properly… I'm sure there's other ways I could minimise my emissions but it wouldn't be through this.

(P8, male, 41, well‐controlled, DPI ICS‐formoterol).

Others raised manufacturers' responsibility to develop environmentally friendly alternatives to pMDIs:

Why aren't the Ventolin manufacturers swapping over to the different type of container?

(P14, female, 67, not well‐controlled, pMDIs)

I think it should be known more. Think that maybe the drug companies can change things as well. You know, it's got to come from manufacturers as well.

(P2, female, 62, partially controlled, SMI)

And rightly or wrongly, I'm not going to change what my puffers are…So it's not that I don't care for the environment because I do a lot of things for the environment. But I'll put that one back on the drug companies.

(P22, female, 67, not well‐controlled, DPI and pMDI)

### 3.4 Influence of Fear, Familiarity and Ease of Use on Preferences and Willingness to Change

Despite most participants scoring in the range of partially or not well‐controlled asthma, many were satisfied with their current treatment routine and reluctant to change unless experiencing an exacerbation in symptoms. This was often attributed to the mental load of navigating uncertainty, learning new routines, or risking ineffective symptom control with another inhaler.

Every time I change puffers, then I have to go through the whole process of working out how effective it is.

(P22, female, 67, not well‐controlled, DPI SABA, pMDI ICS‐formoterol and LABA)

That's what I like about it. I know I've got the confidence in it. I can use it and it works and it's all good and I can just carry on and forget I've got asthma.

(P7, female, 44, not well‐controlled, pMDI SABA, DPI ICS‐formoterol)

Several participants expressed interest and willingness to considering a discussion on changing devices or their asthma management with their health care provider, but only if safe and effective equivalents were available:

Only if I knew that there was a reasonable alternative that still gave me breath. But the reality is I don't know of anything that works like the Ventolin does.

(P7, female, 44, not well‐controlled asthma, pMDI SABA, DPI ICS‐formoterol)

For some participants prior negative experiences with DPIs, or more theoretical uncertainty about the effectiveness of ICS‐formoterol or SABA DPIs in emergencies, contributed to scepticism and unwillingness to change. Especially, SABA pMDI users valued the physical sensation of immediate relief from their inhaler and expressed scepticism that medication from DPIs *‘doesn't go inside’* (P5) *or* could not *‘force[…] the dose into your lungs’* (P15).

I had to call the ambulance because I couldn't breathe after taking that powdered one last time… because of that traumatic incident, I'm not really keen to try it again.

(P9, female, 48, partially controlled, pMDI SABA and ICS‐formoterol)

I wonder if I was having an attack if I would be able to breathe in powder? And not cough more and cause more of a problem.

(P15, female, 49, partially controlled, pMDI SABA, DPI ICS‐LABA)

There's something about that propellant that when you're having an attack, that you know, it seems to take the edge off of it, or faster or something. So, I'd be really hesitant to get rid of that and move entirely to the powder for an attack, you know, when you've got asthma.

(P13, female, 41, well‐controlled, pMDI SABA, DPI ICS‐formoterol)

You know, like, when you're feeling bad and you need help, trying to suck in really hard to get your medication is not really what you want to be doing.

(P9, female, 48, partially controlled, pMDI SABA and ICS‐formoterol)

Overall, participants held strong preferences for devices that were easy to use, familiar, and provided a psychological sense of comfort. These factors were key drivers of continued inhaler patterns and unwillingness to consider alternative options.

Familiarity. (…) ‐ I know what it does. Particularly mentally now; I've been taking it [Ventolin] since I was a kid.

(P19, female, 52, not well‐controlled asthma, pMDI SABA and DPI ICS‐LABA)

Before those stressful moments when you are having an attack, you want something there that you know works, and you've had your whole life.

(P13, female, 41, well‐controlled, DPI ICS‐formoterol and pMDI SABA)

Many participants described themselves as long‐term users of a certain inhaler ‐ *‘creature(s) of habit’* (P13, female, 41, well‐controlled, pMDI SABA) – sometimes since their childhood diagnosis, which contributed to the belief that their inhaler was reliable and effective, almost a *‘security blanket’* (P13). This attachment seemed particularly strong in continued pMDI SABA users:

I've had it [Ventolin] for a while, so sometimes you get used to certain things…been helping me for the last 15 odd years, so I will stick to it.

(P5, female, 75, partially controlled, pMDI SABA and ICS‐LABA)

Even participants not actively using SABA relievers still purchased or kept them on hand *‘just in case’* (P8, male, 41, well‐controlled) for reassurance. In some cases, psychological comfort superseded GP recommendations:

He [GP] was like this one, try this, it's better, and he sold it to me as like, then you don't need the Ventolin, just use this for everything. But then I was like, yeah, I'm still gonna keep the salbutamol [SABA]. (…) I bring it every time I leave the house (…) even though the chances of needing it are so low for me. It's really psychological knowing that it's there and that you'll be okay.

(P13, female, 41, well‐controlled asthma, DPI ICS‐formoterol and pMDI SABA)

Participants were generally aware of the guideline recommendation not to use SABA inhalers *‘more than occasionally’*, with most appreciating the importance of regular preventer or maintenance treatment as part of their asthma management. However, most still used SABA relievers, even if also using AIR‐only or MART inhalers, and some participants felt that these evidence‐based recommendations against SABA overuse were too general for their individual circumstances, relying on their doctor's guidance instead:

I think it's difficult to generalise something like that. So I kind of take it with a grain of salt. I'm very much more guided by my medical team. Yeah, than a general statement.

(P7, female, 44, not well‐controlled, ICS‐formoterol and SABA)

Some rejected this generalised messaging on SABA overuse based on their personal lifelong positive experiences with instant relief:

I don't know that it's a hundred percent true. Because Ventolin has always helped me…I'm still sceptical that that's true…every time I use it, I feel relief. So, I haven't experienced it, not working for me…I'm 45 and I've been using Ventolin my whole life.

(P17, female, 45, not well‐controlled, ICS‐LABA‐LAMA and SABA)

### 3.5 The Role of Health Professionals and Access to Care and Information

Health professionals played a central role in shaping participants' inhaler use and broader asthma management. Across the interviews, physician recommendation, GP or specialist, was the primary factor that determined which inhalers participants used and if they considering changes to their asthma management. Many participants described not questioning their care or seeking alternatives: *‘I just do as I'm told’* (P17, female, 45, not well‐controlled).

While some participants felt confident in managing their asthma ‐ *‘I know my lungs’* (P8, male, 41, well‐controlled) ‐ most ultimately deferred to medical expertise:

Well, I assume it was just the GP changing things for me. It's not anything I would have said, I don't want this or I'm having issues.

(P18, female, 62, well‐controlled)

I know my body best, but the medical professionals know my medication best. My treatment best.

(P19, female, 52, not well‐controlled)

For many, this meant that any change in inhaler type or management plan would only be considered if explicitly recommended by their doctor:

I wouldn't change to a dry powder inhaler, or I wouldn't change what I'm doing unless I had medical advice.

(P22, female, 67, not well‐controlled asthma)

At the same time, several participants described managing their SABA‐use autonomously, particularly during flare‐ups, sometimes by‐passing their doctor altogether.

They say see your GP but I'll work it out.

(P5, female, 75, partially controlled, SABA and ICS‐LABA)

I was changing GPs, and I told him that I'd been using my Ventolin several times a day. And he went into this full‐blown panic mode. And I was like dude I've been doing it my whole life. It's okay don't stress. And he was like, ah!

(P17, female,45, not well‐controlled, SABA and ICS‐LABA‐LAMA)

Participants had mixed views about whether health professionals should raise environmental considerations during asthma care. Some felt it was their responsibility to include environmental information in discussion,

I think the person that's provided the information at that first point of contact should be the person that's kind of like passing it on.

(P21, female, 37, partially controlled)

but others were sceptical about whether GPs had the time or knowledge to cover this during routine consultations:

I doubt a GP would go into this kind of detail…I'm a bit sceptical as to whether GPs would really explain the differences and the benefits and the downsides of all these things.

(P16, female, 34, partially controlled)

Several participants expressed interest in discussing alternatives with their doctor in the future:

I know I'm gonna be discussing this with my doctor. Because, yeah, anything that saves the planet has to be good. So as long as the alternate inhaler works and is easy to use.

(P4, female, 73, not well‐controlled)

However, for most, environmental concerns alone were not strong enough to prompt them to initiate a conversation:

Personally, for me, I wouldn't make an appointment, particularly because I know the doctors are so busy to just change over on the basis of environmental impact.

(P1, female, 45, partially controlled)

Many participants described challenges in accessing information and support needed to enable them to effectively manage their condition. This resulted in knowledge gaps around optimal management options and specific inhaler use techniques as well as limited awareness around available device types. Some participants were reluctant to see a GP or specialist for asthma management and review consultations due to the high costs, wait lists, and time required. Several felt that these barriers to care contributed to over‐reliance on pMDI SABA inhalers:

I think it's very true that people often overuse Ventolin inhalers. Particularly…in exacerbation, as opposed to going to the doctor, because, what's the doctor going to say, just increase this. And the cost of a consultation, on top of the cost of another inhaler, or something else.

(P20, female, 44, not well‐controlled, ICS‐LABA, SABA)

Couldn't get into the doctors where I lived, rural areas, had to wait six, seven, 8 weeks to get into the doctor. Was shocking.

(P14, female, 67, not well‐controlled, ICS‐LABA‐LAMA, SABA)

Yeah, it's really cost prohibitive to have to book a doctor's appointment for me and the kids individually…Not to mention the time.

(P3, female, 42, partially controlled, ICS‐formoterol, SABA)

As a result, many participants had turned to other sources for information, such as Asthma Australia, asthma care nurses and pharmacists. These were often considered more informative and less rushed:

They [Asthma Australia] did a, (…), kind of a one‐on‐one, a nurse to talk you through the symptoms and what to do and answer all your crazy questions and things like that. So, yeah, it was fantastic. Very helpful.

(P15, female, 49, not well‐controlled)

But the chemist did have to explain how to use it because it was quite, many different things, different process, I guess.(…) They just explained kind of how to use it and I think they even had a practice one in the chemist.

(P15, female, 49, partially controlled)

No one in all those years had ever really sat down and explained (…) what asthma was. But looking at the way that they [asthma nurse educator] use the diagrammatic models and everything really helped you understand exactly what goes on inside when you're having an asthma attack and everything. Yeah, it was super interesting.

(P1, female, 45 partially controlled)

## 4 Discussion

### 4.1 Principal Findings

Participants had limited awareness of inhaler types and their environmental impact, with many surprised by the emissions from pMDIs. While environmental sustainability was valued, fast‐acting SABA pMDIs were prioritised due to perceptions of effectiveness, familiarity, and psychological reassurance during acute attacks. Clinicians strongly influenced inhaler choices, with most participants relying on their advice and expressing limited decision‐making autonomy. Barriers such as cost, time, and rural location contributed to knowledge gaps and potential overreliance on SABAs. Positive experiences with asthma care nurses and pharmacists suggest opportunities to enhance support and education.

### 4.2 Findings in Relation to Other Studies

Our findings align with previous research indicating that while patients often express concern for the environment, this is not enough motivation for changes in inhaler use by itself. Previous surveys have found that although many patients value sustainability, fewer are willing to switch devices [16, 32]. For example, a New Zealand cross‑sectional survey found that patients' willingness to consider switching to lower‑emission inhalers was conditional on reassurance regarding efficacy, ease of use and symptom control, with environmental impact ranked as a secondary consideration [16]. Similarly, a large multinational survey of patients and healthcare professionals across 42 countries reported that while sustainability was viewed positively, inhaler choice was predominantly driven by perceived effectiveness, familiarity and clinical reassurance, with environmental impact consistently ranked among lower priorities [32]. Our study helps explain this gap, showing that familiarity, psychological comfort and perceived efficacy of SABA pMDIs are main drivers of patient preference; consistent with prior work identifying these factors as key contributors to SABA overreliance [33].

Unlike earlier studies where patients were less aware of the risks of SABA overuse and inconsistent in their preventer use [33], many of our participants reported relatively good preventer adherence and a general sense that frequent SABA use was undesirable. However, this awareness was often vague, without clear expressions of understanding of why, or how risks such as worsening asthma control, increased exacerbations or mortality arise with overuse. Participants also did not seem to consistently apply this to their own situation. In practice, many distinguished between ‘overuse’ in principle and their own SABA use, which was commonly justified as necessary for reassurance or rapid, reliable symptom relief. This helps explain how awareness of harm and reported preventer adherence can coexist with continued SABA reliance and suboptimal asthma control, suggesting that while awareness may be improving, fully informed choice is still lacking and implementation of best‐practice guidelines remains inconsistent or is undermined by over‐the‐counter SABA purchases. Similar concerns have been raised in Australian population studies showing high rates of uncontrolled asthma and SABA overuse despite updated national guidelines [5, 34, 35].

The role of health professionals, particularly GPs, in shaping inhaler use is well documented [36, 37]. Our findings reinforce that many patients defer to clinician advice but often lack opportunities for in‐depth consultations, echoing concerns about time constraints and fragmented care raised in prior studies [38]. Shared and informed decision‐making is essential for safe and consensual inhaler type and/or medication switching [10, 36, 37]. Our study supports this, showing that patients are open to change when reassured of efficacy and safety. While this study focused on patient perspectives, understanding how these intersect with clinician views and prescribing practices will be important for future research and implementation.

Efforts to reduce emissions through optimal asthma care, which can improve asthma symptoms and exacerbations, consequently reducing need for reliever use, emergency treatment and avoidable hospitalisations, are gaining traction globally [15]. In the UK, the SENTINEL trial achieved sustained improvements in asthma outcomes and reductions in SABA overuse, with carbon footprint reductions as a co‐benefit. This was achieved through co‑designed, guideline‑concordant interventions that prioritised shared decision‑making, targeted reviews and reassurance about efficacy and safety, rather than through device substitution alone [39]. On the other hand, evidence from the US Veterans Health Administration highlights that mandatory inhaler switches ‐ whether driven by clinical, cost or environmental concerns ‐ risk undermining trust and adherence. If patients' concerns are not adequately addressed there are potential consequences for asthma control [10]. Our study results align with these findings, with patients expressing strong attachment to familiar ‘lifesaving’ inhalers, concerns about efficacy and safety of nonfamiliar inhalers and reliance on clinician reassurance. Changing inhalers is most likely to be successful when patients are actively engaged and shared decision‐making addresses their fears and priorities, rather than through an isolated focus on the device alone [10, 14, 40]. Thus, reinforcing the principle that the ‘greenest’ inhaler is ultimately the one a person knows how to use correctly and will adhere to [37].

Additionally, many of our participants held inhaler manufacturers responsible for environmental impact reductions. As of 2026, nine pharmaceutical companies have low‐emission pMDIs in late‐stage development, with initial regulatory approvals and early launches just beginning [41]. It remains to be seen how affordable and accessible these will be and how long it will take for regulators to approve them [10]. In combination, these strategies are consistent with the Australian National Sustainable Asthma Care Roadmap Roundtable Report, which advocates for an integrated whole‐of‐systems approach to achieving high‐quality, low‐carbon asthma care delivery [3].

### 4.3 Implications for Policy, Practice and Research

Our findings suggest that improving asthma care and supporting more sustainable inhaler use in Australia is likely to require coordinated action across policy, clinical practice and research, informed by patient perspectives and existing evidence.

Given participants' limited awareness of potential harms from SABA overuse and reliance on relievers, mechanisms to monitor and address excessive SABA prescribing and over‐the‐counter purchasing, warrant consideration at a policy level. Consistent with participants' frequent interactions with pharmacists, prompts for pharmacist‐initiated reviews could help identify patients at risk of poor asthma control and encourage referral for asthma management reviews. While our study did not assess the effectiveness of subsidised or targeted asthma reviews for patients with high SABA use, these could support transitions to evidence‐based care. However, barriers to care identified in this study such as cost, time and access constraints would need to be addressed. Introducing carbon footprint labelling for inhalers may enhance patient awareness and enable clinicians and patients to consider environmental impact in treatment decisions. However, its effectiveness in influencing decision‐making remains uncertain and should be evaluated alongside other education strategies.

In clinical practice, our findings highlight several opportunities to reduce low‐value, high‐carbon care. Firstly, through ensuring accurate asthma diagnosis prior to prescribing [37]. A recent systematic review found between 17% to 66% of diagnoses being overturned upon subsequent review [42]. In patients with confirmed diagnosis, shared decision‐making to support safe and acceptable transitions to guideline‐recommended treatments needs to be prioritised. Our findings show that many patients rely heavily on clinician advice but face barriers to accessing high quality asthma care and information, limiting their awareness of alternatives. GPs, pharmacists and asthma educators are therefore well‐positioned to provide accessible information, reinforce inhaler technique and support informed decision‐making. Consistent with participants' emphasis on early treatment experiences, prescribing decisions made at diagnosis may be particularly influential, underscoring the importance of initiating evidence‐based asthma care, including AIR‐only and MART regimens and considering prescribing of one of the widely available DPI options, which have a carbon footprint of less than 5% of pMDIs [10, 43, 44], when clinically appropriate and suitable for patient abilities. Consistent messaging during hospital admissions and discharge planning also offers opportunities to reinforce best‐practice care.

Future research should focus on co‐designing and evaluating implementation strategies to support uptake of AIR‐only and MART therapies and reduce reliance on SABA, as per the Australian Asthma Handbook guidelines [9]. Given that this study did not explore clinician perspectives or test specific interventions, collaborating with clinicians and patients will be essential to develop practical, acceptable strategies that account for real‐world constraints. Embedding asthma action plans, decision‐support tools and education resources into clinical and pharmacy systems may streamline care while further research should assess the acceptability and effectiveness of approaches such as labelling or pharmacist‐led interventions. This will be critical to evaluate and tailor strategies to diverse Australian contexts and accelerate delivery of high quality, low‐carbon asthma care.

### 4.4 Strengths and Limitations

Our study provides in‐depth insights into the perspectives of adult asthma patients from metropolitan, regional and rural areas, across varying disease severity. Participants used a broad range of inhalers and medications, providing a nuanced view of patient experiences and preferences within an Australian sample.

Our study has some limitations. The sample was predominantly female, did not include individuals under the age of 35 and was primarily recruited through Asthma Australia, which may have introduced a selection bias towards more health literate and informed individuals. Health literacy is often associated with capacity to engage in shared decision‐making [45]. As such, high education and health literacy levels in our sample may be reflected in our findings of relatively high willingness and capacity to engage with environmental information. However, higher health literacy does not necessarily translate into a preference for active involvement in all treatment decisions; patients may still prefer to rely on trusted clinicians [46, 47]. In our sample, this may explain why participants appeared both relatively well‐informed and strongly deferential to their clinician. Most participants rarely purchased over‐the‐counter inhalers, suggesting regular opportunities for asthma consultations at prescription renewals. Consequently, the study may not capture the perspectives of patients with less regular engagement in care, who may be more at risk of limited preventer use and high SABA reliance. This is important given that lower health literacy is associated with poorer asthma outcomes, including incorrect inhaler use and reduced adherence [48], following a clear socioeconomic gradient [49]. As such, barriers to guideline‐concordant, high‐quality treatment may be even greater in less health‐engaged populations. Nevertheless, our findings highlight persistent barriers to high‐quality care, even among a relatively informed group. Another limitation to our study is that we did not collect data on participants' Indigenous status and therefore cannot determine whether these perspectives are represented in our sample. Aboriginal and Torres Strait Islander peoples experience a higher burden of asthma and may have distinct experiences of care and medication use [50]. As such, the findings may not reflect the perspectives of these communities and should be interpreted with caution in this respect. Ensuring that future research includes Aboriginal and Torres Strait Islander perspectives will be important to better understand how these issues are experienced across the Australian population.

## 5 Conclusions

While environmental concern matters to patients, it alone is insufficient to drive changes in asthma management, which is shaped by habit, familiarity and fear of exacerbations. Supporting implementation of evidence‐based care requires clinician leadership, accessible information and trusted alternatives that can improve asthma control while reducing emissions.

## Author Contributions

**Imogen Le Couteur:** investigation, writing – original draft, methodology, writing – review and editing, project administration, data curation, formal analysis. **Sean Docking:** conceptualisation, funding acquisition, writing – review and editing, software. **Katy Bell:** conceptualisation, funding acquisition, supervision, writing – review and editing. **Alexandra Barratt:** conceptualisation, funding acquisition, writing – review and editing, supervision. **Rebecca Haddock:** writing – review and editing. **Darlene Cox:** conceptualisation, funding acquisition, writing – review and editing. **Kate Wylie:** conceptualisation, funding acquisition, writing – review and editing. **Luise Kazda:** conceptualisation, data curation, supervision, validation, visualisation, writing – original draft, writing – review and editing, funding acquisition, formal analysis, project administration, resources, software.

## Ethics Statement

Ethics approval was granted from Monash University Human Research Ethics Committee (2024/MUHREC44951). All potential participants were provided with a participant information statement, detailing how the collected data will be used. Informed consent for publication was sought and obtained from all participants prior to their involvement in the study.

## Conflicts of Interest

The authors declare no conflicts of interest.

## Supporting information

Supporting File 1:

Supporting File 2:

Supporting File 3:

Supporting File 4:

## Data Availability

The data that support the findings of this study are available on request from the corresponding author. The data are not publicly available due to privacy or ethical restrictions.

## References

[1] S. M. Jayasooriya , G. Devereux , J. B. Soriano , et al., “Asthma: Epidemiology, Risk Factors, and Opportunities for Prevention and Treatment,” Lancet Respiratory Medicine 13, no. 8 (2025): 725–738, 10.1016/S2213-2600(24)00383-7. 40684789

[2] Australian Bureau of Statistics , Asthma 2023, https://www.abs.gov.au/statistics/health/health-conditions-and-risks/asthma/2022.

[3] M. Forrester , C. Needham , S. Allender , et al., The National Sustainable Asthma Care Roadmap – Roundtable Report (2024).

[4] L. Kazda , A. L. Barratt , S. Docking , et al., “Estimated Carbon Emissions for Pbs‐Subsidised Prescription Respiratory Inhalers, Australia, 2019‐2023: A Descriptive Analysis,” Medical Journal of Australia 223 (2025): 214–217, 10.5694/mja2.52715. 40567109 PMC12358100

[5] H. K. Reddel , S. M. Sawyer , P. W. Everett , P. V. Flood , and M. J. Peters , “Asthma Control in Australia: A Cross‐Sectional Web‐Based Survey in a Nationally Representative Population,” Medical Journal of Australia 202, no. 9 (2015): 492–496, published Online First: 2015/05/15 [10.5694/mja14.01564. 25971575

[6] B. I. Nwaru , M. Ekström , P. Hasvold , F. Wiklund , G. Telg , and C. Janson , “Overuse of Short‐Acting β2‐agonists in Asthma Is Associated With Increased Risk of Exacerbation and Mortality: A Nationwide Cohort Study of the Global Sabina Programme,” European Respiratory Journal 55, no. 4 (2020): 1901872, published Online First: 2020/01/18 [10.1183/13993003.01872-2019. 31949111 PMC7160635

[7] Global Initiative for Asthma , Global Strategy for Asthma Management and Prevention, 2023.

[8] National Asthma Council , New guidelines state SABA inadequate treatment for asthma 2025, accessed October 16, 2025, https://www.nationalasthma.org.au/news/2025/new-guidelines-state-saba-inadequate-treatment-for-asthma.

[9] National Asthma Council , Australian Asthma Handbook 2025, accessed October 16, 2025, https://www.asthmahandbook.org.au/.

[10] M. Alkhunaizi , N. Nassikas , L. E. Eggert , and J. Tirumalasetty , “Reducing Inhaler‐Related Greenhouse Gas Emissions,” Journal of the American Medical Association 333 (2025): 2051, published Online First: 2025/05/01. 10.1001/jama.2025.5942. 40310611

[11] M. J. Loftus , M. S. Cumpston , S. Barnes , et al., “Efficacy and Safety of Different Inhaler Types for Asthma and Chronic Obstructive Pulmonary Disease,” A Systematic Review and Meta‐Analysis. medRxiv preprint 36 (2026): 18, 10.1038/s41533-026-00488-4. PMC13022311 41698939

[12] R. De Sain , A. Irwin , A. McGushin , et al., Estimates of Australian Health System Greenhouse Gas Emissions (Australian Centre for Disease Control, 2024), 2021–2022.

[13] United States Environmental Protection Agency , Greenhouse Gas Equivalencies Calculator, accessed October 17, 2025, 2024, https://www.epa.gov/energy/greenhouse-gas-equivalencies-calculator.

[14] A. J. K. Wilkinson , E. Maslova , C. Janson , et al., “Greenhouse Gas Emissions Associated With Suboptimal Asthma Care in the Uk: The Sabina Healthcare‐Based Environmental Cost of Treatment (Carbon) Study,” Thorax 79, no. 5 (2024): 412–421, 10.1136/thorax-2023-220259. 38413192 PMC11041603

[15] A. S. Rabin , J. Tirumalasetty , and S. I. Maximous , “Rethinking Inhalers in the Era of Climate Change,” Journal of the American Medical Association 334 (2025): 1623, 10.1001/jama.2025.17819. 41051784

[16] R. Hancox , M. Woodall , J. Ma , et al., “Investigating Attitudes and Insights into the Global Warming Impact of Inhalers,” New Zealand Medical Journal 136, no. 1573 (2023): 94–105. 10.26635/6965.6026 37054459

[17] E. Rothwell , J. McElvaney , A. Fitzpatrick , et al., “Evaluating Inhaler Technique, Patient Preferences and Opportunities for Improvement in Hospitals in the Uk,” Future Healthcare Journal 11, no. 2 (2024): 100141, 10.1016/j.fhj.2024.100141. 38845621 PMC11153909

[18] A. Hodge , H. Wickham , K. Florman , et al., “The Patient Perspective on the Environmental Impact of Inhalers,” Respiratory Medicine 235 (2024): 107864, 10.1016/j.rmed.2024.107864. 39566646

[19] D. Quantz , G. Y. C. Wong , and K. Liang , “Patient Perspectives on the Environmental Impact of Inhalers: A Survey in British Columbia,” Canadian Pharmacists Journal Revue des Pharmaciens du Canada 156, no. 6 (2023): 298–302, 10.1177/17151635231202980. 38024456 PMC10655797

[20] National Asthma Council , Reducing the Environmental Impact of Asthma Treatment (National Asthma Council Australia, 2024).

[21] Department of Health and Aged Care , National Health and Climate Strategy Canberra: Australian Government, Department of Health and Aged Care; 2023 [updated December 2023, accessed February 27, 2025.https://www.health.gov.au/sites/default/files/2023-12/national-health-and-climate-strategy.pdf.

[22] Asthma Australia , Understanding the Sustainable Asthma Care Roadmap 2025, accessed May 14, 2026. https://asthma.org.au/blog/quality-and-sustainability-in-asthma-care/.

[23] M. J. Crotty , The Foundations of Social Research: Meaning and Perspective in the Research Process (Routledge, 1998), 10.4324/9781003115700.

[24] A. Tong , P. Sainsbury , and J. Craig , “Consolidated Criteria for Reporting Qualitative Research (Coreq): a 32‐item Checklist for Interviews and Focus Groups,” International Journal for Quality in Health Care 19, no. 6 (2007): 349–357, 10.1093/intqhc/mzm042. 17872937

[25] E. F. Juniper , P. M. O′byrne , G. Guyatt , P. Ferrie , and D. King , “Development and Validation of a Questionnaire to Measure Asthma Control,” European Respiratory Journal 14, no. 4: 902–907, 10.1034/j.1399-3003.1999.14d29.x. 10573240

[26] E. F. Juniper , J. Bousquet , L. Abetz , and E. D. Bateman , “Identifying ‘Well‐Controlled’ and ‘Not Well‐Controlled’ Asthma Using the Asthma Control Questionnaire,” Respiratory Medicine 100, no. 4 (2006): 616–621, 10.1016/j.rmed.2005.08.012. 16226443

[27] L. D. Chew , J. M. Griffin , M. R. Partin , et al., “Validation of Screening Questions for Limited Health Literacy in a Large VA Outpatient Population,” Journal of General Internal Medicine 23, no. 5 (2008): 561–566, 10.1007/s11606-008-0520-5. 18335281 PMC2324160

[28] J. Ritchie and L. Spencer , “Qualitative Data Analysis for Applied Policy Research.” in Analyzing Qualitative Data, eds. A. Bryman and R. Burgess (Routledge, 1994), 173–194.

[29] NVivo Qualitative Data Analysis Software , [program], 15 version. Denver, CO: Lumivero, 2023.

[30] Microsoft Excel for Microsoft 365 [program] (Microsoft Corporation, 2025).

[31] National Asthma Council , Asthma Action Plans 2024, accessed October 20, 2025. https://www.nationalasthma.org.au/health-professionals/asthma-action-plans.

[32] G. Lough , S. Bosnic‐Anticevich , N. Roche , and O. S. Usmani , “Patient and Provider Perspectives Driving Inhaler Choice,” Chest 166, no. 5 (2024): 934–937, published Online First: 2024/07/05. 10.1016/j.chest.2024.06.3774. 38964671

[33] S. Blakeston , G. Harper , and J. Zabala Mancebo , “Identifying the Drivers of Patients' Reliance on Short‐Acting β2‐agonists in Asthma,” Journal of Asthma 58, no. 8 (2021): 1094–1101, published Online First: 2020/05/30. 10.1080/02770903.2020.1761382. 32469667

[34] E. D. Bateman , D. B. Price , H. C. Wang , et al., “Short‐Acting β2‐agonist Prescriptions Are Associated With Poor Clinical Outcomes of Asthma: The Multi‐Country, Cross‐Sectional Sabina Iii Study,” European Respiratory Journal 59, no. 5 (2022): 2101402, published Online First: 2021/09/26. 10.1183/13993003.01402-2021. 34561293 PMC9068976

[35] E. A. Azzi , V. Kritikos , M. J. Peters , et al., “Understanding Reliever Overuse in Patients Purchasing Over‐The‐Counter Short‐Acting beta(2) Agonists: An Australian Community Pharmacy‐Based Survey,” BMJ Open 9, no. 8 (2019): e028995, published Online First: 2019/08/16. 10.1136/bmjopen-2019-028995. PMC6701672 31412998

[36] S. Doyle , A. Lloyd , A. Williams , et al., “What Happens to Patients Who Have Their Asthma Device Switched Without Their Consent?,” Primary Care Respiratory Journal 19, no. 2 (2010): 131–139, published Online First: 2010/02/23. 10.4104/pcrj.2010.00009. PMC6602231 20174771

[37] B. D. Montgomery and J. D. Blakey , “Respiratory Inhalers and the Environment,” Australian Journal of General Practice 51, no. 12 (2022): 929–934, 10.3316/informit.786547947693053. 36451327

[38] I. Hesso , R. Kayyali , and S. Nabhani‐Gebara , “Supporting Respiratory Patients in Primary Care: A Qualitative Insight From Independent Community Pharmacists in London,” BMC Health Services Research 19, no. 1 (2019): 5, published Online First: 2019/01/07 [10.1186/s12913-018-3814-2. 30611264 PMC6321650

[39] M. G. Crooks , L. Crowther , H. Cummings , et al., “Improving Asthma Care Through Implementation of the Sentinel Programme: Findings From the Pilot Site,” ERJ Open Research 9, no. 3 (2023): 00685‐2022, published Online First: 2023/05/25. 10.1183/23120541.00685-2022. PMC10204732 37228273

[40] M. G. Crooks , H. Cummings , A. H. Morice , et al., “Reducing Short‐Acting Beta‐Agonist Use in Asthma: Impact of National Incentives on Prescribing Practices in England and the Findings From Sentinel Plus Early Adopter Sites,” NPJ Primary Care Respiratory Medicine 34, no. 1 (2024): 6, published Online First: 2024/04/30. 10.1038/s41533-024-00363-0. PMC11058200 38684652

[41] United Nations Environment Programme (UNEP) , *Montreal Protocol on Substances that Deplete the Ozone Layer*, Report of the Technology and Economic Assessment Panel: Progress Report, May 2025, https://ozone.unep.org/science/assessment/teap,ISBN:978-9914-733-73-0.

[42] S. Sanders , A. Barratt , R. Buchbinder , et al., “Evidence for Overdiagnosis in Noncancer Conditions Was Assessed: A Metaepidemiological Study Using the ‘Fair Umpire’ Framework,” Journal of Clinical Epidemiology 165 (2024): 111215, published Online First: 2023/11/13. 10.1016/j.jclinepi.2023.11.005. 37952702

[43] C. Janson , R. Henderson , M. Löfdahl , M. Hedberg , R. Sharma , and A. J. K. Wilkinson , “Carbon Footprint Impact of the Choice of Inhalers for Asthma and Copd,” Thorax 75 (2020): 82–84. 31699805 10.1136/thoraxjnl-2019-213744 PMC6929707

[44] A. Woodcock , K. M. Beeh , H. Sagara , et al., “The Environmental Impact of Inhaled Therapy: Making Informed Treatment Choices,” European Respiratory Journal 60, no. 1 (2022): 2102106, published Online First: 2021/12/18. 10.1183/13993003.02106-2021. 34916263 PMC9301054

[45] D. M. Muscat , H. L. Shepherd , D. Nutbeam , L. Trevena , and K. J. McCaffery , “Health Literacy and Shared Decision‐Making: Exploring the Relationship to Enable Meaningful Patient Engagement in Healthcare,” Journal of General Internal Medicine 36, no. 2 (2021): 521–524, published Online First: 2020/05/31. 10.1007/s11606-020-05912-0. 32472490 PMC7878628

[46] I. Pokhilenko , T. E. M. van Esch , A. E. M. Brabers , and J. D. de Jong , “Relationship Between Trust and Patient Involvement in Medical Decision‐Making: A Cross‐Sectional Study,” PLoS One 16, no. 8 (2021): e0256698, 10.1371/journal.pone.0256698. 34437626 PMC8389380

[47] A. L. Caress , K. Luker , A. Woodcock , and K. Beaver , “A Qualitative Exploration of Treatment Decision‐Making Role Preference in Adult Asthma Patients,” Health Expectations 5, no. 3 (2002): 223–235, published Online First: 2002/08/30. 10.1046/j.1369-6513.2002.00181.x. 12199661 PMC5060151

[48] A. D. Federman , J. P. Wisnivesky , M. S. Wolf , H. Leventhal , and E. A. Halm , “Inadequate Health Literacy Is Associated With Suboptimal Health Beliefs in Older Asthmatics,” Journal of Asthma 47, no. 6 (2010): 620–626, 10.3109/02770901003702816. 20636188

[49] N. D. Berkman , S. L. Sheridan , K. E. Donahue , D. J. Halpern , and K. Crotty , “Low Health Literacy and Health Outcomes: An Updated Systematic Review,” Annals of Internal Medicine 155, no. 2 (2011): 97–107, 10.7326/0003-4819-155-2-201107190-00005. 21768583

[50] AIHW , First Nations People With Asthma 2023 (AIHW, Australian Government, 2023).

