# Supplementary material for: Managing Asthma Well and Sustainably – Patient Perspectives Explored
Source: Health Expect. 2026 Jul 3;29(4):e70751. doi: 10.1111/hex.70751 (PMC13332319; doi:10.1111/hex.70751)
Supplement: Supplementary file 1 — Supporting File 1: [file HEX-29-e70751-s003.docx]

**S1. COREQ Checklist^1^**

| **No. Item** | **Guide questions/description** | | **Notes** |
| --- | --- | --- | --- |
| **Domain 1: Research team and reflexivity** | | | |
| *Personal Characteristics* | | | |
| 1. Interviewer | Which author/s conducted the interviews? | IL conducted all interviews with participants | |
| 1. Credentials | What were the researcher’s credentials? | IL: BSc Hons (Psyc) | |
| 1. Occupation | What was their occupation at the time of the study? | IL was employed as a Research Assistant with The University of Sydney | |
| 1. Gender | Was the researcher male or female? | The interviewer (IL) is female | |
| 1. Experience and training | What experience or training did the researcher have? | IL has a background in psychology and experience and training in using qualitative research methods. | |
| *Relationship with Participants* | | | |
| 1. Relationship established | Was a relationship established prior to study commencement? | IL did not have contact with participants prior to study commencement | |
| 1. Participant knowledge of the interviewer | What did the participants know about the researcher? E.g. personal goals, reasons for doing the research | Participants were advised that the study aimed to investigate people’s knowledge, awareness, and attitudes towards respiratory inhalers and environmentally sustainable care. Participants did not have any other knowledge about IL. | |
| 1. Interview characteristics | What characteristics were reported about the interviewer/ facilitator? E.g. Bias, assumptions, reasons and interests in the research topic | Participants were informed IL worked as a Research Assistant at The University of Sydney, and was aiming to explore their knowledge, awareness, and attitudes towards respiratory inhalers and environmentally sustainable care. | |
| **Domain 2: Study design** | | | |
| *Theoretical Framework* | | | |
| 1. Methodological orientation and theory | What methodological orientation was stated to underpin the study? e.g. grounded theory, discourse analysis, ethnography, phenomenology, content analysis | This study followed a framework analysis methodology. Analysis followed an inductive approach to ensure conclusions were drawn from the data. | |
| *Participant Selection* | | | |
| 1. Sampling | How were participants selected? e.g.  purposive, convenience, consecutive,  snowball | Participants were recruited through a newsletter and social media advertisements shared consumer advocacy organisations aimed at recruiting a diverse sample of asthmatics across Australia. They were selected as a convenience sample. | |
| 1. Method of approach | How were participants approached? Eg. face-to-face, telephone, mail, email | Participants responded to the study advertisement by completing the online Qualtrics consent form and pre-interview questionnaire. Eligible participants were then contacted via response email to arrange an interview. | |
| 1. Sample size | How many participants were in the study? | 23 participants were in the study. | |
| 1. Non-participation | How many people refused or dropped out? | No participants dropped out of the study after consenting to participate and undertaking the interview. Four participants declined participating in an interview after contact was made due to time constraints. 3 candidates did not respond to the interview invitation. | |
| *Setting* | | | |
| 1. Setting of data collection | Where was the data collected? E.g. home, clinic, workplace | Interviews were conducted via Zoom or telephone at a time mutually agreeable to participants and the researcher. | |
| 1. Presence of non-participants | Was anyone else present besides the participants and researchers? | Only the participant and researcher (IL) were present at the time of the interview. | |
| 1. Description of sample | What are the important characteristics of the sample? E.g. demographic data, date | Interviews were conducted between November 2024 and January 2025. See Table 1 for demographic data. | |
| *Data Collection* | | | |
| 1. Interview guide | Were questions, prompts, guides provided by the authors? Was it pilot tested? | See supplementary material for interview guide and one visual prompt. The guide was not provided to the participants; the prompt was shown during the interview. The interview was pilot tested by one person diagnosed with asthma. | |
| 1. Repeat interviews | Were repeat interviews carried out? If yes, how many? | No repeat interviews were conducted. | |
| 1. Audio/visual recording | Did the research use audio or visual recording to collect the data? | All interviews were audio recorded. | |
| 1. Field notes | Were field notes made during and/or after the interview? | Field notes were made during and after the interviews. | |
| 1. Duration | What was the duration of the interviews? | Interviews went for an average time of 37 minutes. Interview duration ranged from 25-54 minutes. | |
| 1. Data saturation | Was data saturation discussed? | Data saturation was agreed upon by IL and LK and reached for all major themes and concepts. | |
| 1. Transcripts returned | Were transcripts returned to participants for comment and/or correction? | Transcripts were not returned to participants. | |
| **Domain 3: Analysis and findings** | | | |
| *Data Analysis* | | | |
| 1. Number of data coders | How many coders coded the data? | One researcher (IL) coded all the data, with a second researcher validating a subset of the data (six transcripts). | |
| 1. Description of the coding tree | Did authors provide a description of the coding tree? | Themes and subthemes are detailed in the manuscript. | |
| 1. Derivation of themes | Were themes identified in advance or derived from the data? | Themes were derived from the data following an inductive approach. | |
| 1. Software | What software, if applicable, was used to manage the data? | All qualitative data was coded in NVivo 15. Quantitative data was managed using Excel. | |
| 1. Participant checking | Did participants provide feedback on the findings? | Participants did not provide feedback on the findings. | |
| *Reporting* | | | |
| 1. Quotations presented | Were participant quotations presented to illustrate the themes/ findings? Was each  quotation identified? e.g. participant number | Quotations are presented throughout the results section. These are accompanied by the participant’s identification number, age, gender and asthma control score. | |
| 1. Data and findings consistent? | Was there consistency between the data presented and the findings? | All findings were derived from the data and were supported by participant quotes. | |
| 1. Clarity of major themes | Were major themes clearly presented in the findings? | Major themes are presented in the results section. | |
| 1. Clarity of minor themes | Is there a description of diverse cases or discussion of minor themes? | Diverse cases and minor themes are presented in the results section. | |

1. Tong A, Sainsbury P, Craig J. Consolidated criteria for reporting qualitative research (COREQ): a 32-item checklist for interviews and focus groups. *International Journal for Quality in Health Care* 2007;19(6):349-57. doi: 10.1093/intqhc/mzm042
