# Supplementary material for: Managing Asthma Well and Sustainably – Patient Perspectives Explored
Source: Health Expect. 2026 Jul 3;29(4):e70751. doi: 10.1111/hex.70751 (PMC13332319; doi:10.1111/hex.70751)
Supplement: Supplementary file 2 — Supporting File 2: [file HEX-29-e70751-s001.pdf]

MONASH University

Demographics

What is your name?

____

What is your age (in years)?

____

What is your gender?

- ☐ Woman
- ☐ Man
- ☐ Non-binary / gender diverse
- ☐ __________ My gender identify isn't listed. I identify as:
- ☐ Prefer not to say

What state or territory do you live in?

- ACT
- NSW
- VIC
- QLD
- SA
- TAS
- WA
- NT

What kind of area do you live in?

- Metropolitan (capital cities)
- Regional (small cities/towns)
- Rural
- Remote

Is the main language you speak at home English?

- Yes
- No

What is your highest level of education?

- Postgraduate degree
- Graduate diploma/graduate certificate
- Bachelor degree
- Advanced diploma
- Certificate III/IV
- High school

How confident are you filling out medical forms by yourself?

- Extremely confident
- Quite confident
- Somewhat confident
- A little confident
- Not at all confident

Diagnosis and severity

How long ago were you first diagnosed with asthma?

- Within the last 12 months
- 1 to 5 years ago
- 6 to 10 years ago
- More than 10 years ago

In the past month, how severe would you rate your asthma?

- Mild (mild increased work of breathing, normal respiratory rate, alert and active)
- Moderate (moderate increased work of breathing, increased respiratory rate, active and alert)
- Severe (Markedly increased work of breathing, increased respiratory rate, agitated, pale, other signs of anaphylaxis)

On average, in the last week, how often were you **woken by your asthma** during the night?

- Not at all
- Hardly ever
- A few times
- Several times
- Many times
- A great many times
- Unable to sleep because of asthma

On average, in the last week, how **were your asthma symptoms when you woke up** in the morning?

- No symptoms
- Very mild symptoms
- Mild symptoms
- Moderate symptoms
- Quite severe symptoms
- Severe symptoms
- Very severe symptoms

In general, in the last week, how **limited were you in your day-to-day activities** because of your asthma?

- Not at all limited
- Very slightly limited
- Slighly limited
- Moderately limited
- Very limited
- Extremely limited
- Totally limited

In general, in the last week, how much **shortness of breath** did you experience because of your asthma?

- None
- Very little
- A little
- A moderate amount
- Quite a lot
- A great deal
- An extreme amount

In general, in the last week, how often did you **wheeze**?

- None of the time
- Hardly any of the time
- A little of the time
- A moderate amount of the time
- A lot of the time
- Most of the time
- All the time

Inhaler use

How long have you been using an inhaler/s?

- Less than a 12 months
- 1 to 5 years
- 6 to 10 years
- More than 10 years

Most inhalers available in Australia are either pressurised metered dose inhalers (MDIs) or dry powder inhalers (DPIs). How confident are you that you know the main difference between these two types of inhalers?

- 1 (Not confident at all)
- 2
- 3
- 4
- 5 (Very Confident)

What inhaler/s do you currently use? If you know, please provide details on brand names and whether it is metered dose inhaler or dry powder inhaler.

If unsure, please remember to bring your inhaler to your telephone/online interview

How frequently do you use your inhaler/s?

- Daily
- 4-6 times a week
- 2-3 times a week
- Once a week
- Occasionally

Please indicate if you have used any other inhalers in the past 5 years that you no longer use? If you can remember, please provide details on brand names and whether it is metered dose inhaler or dry powder inhaler.

Do you have an asthma management plan?

- Yes

- No

How regularly is your asthma management plan reviewed?

- Once a year
- Every second year
- Within the last 2-5 years
- It has not been reviewed

Monash University values the privacy of every individual's personal information and is committed to the protection of that information from unauthorised use and disclosure except where permitted by law. For information about the handling of your personal information please see the applicable Statement which applies to you in the context of this survey.

For more information about Data Protection and Privacy at Monash University please see our Data Protection and Privacy Procedure.

Powered by Qualtrics
