# Supplementary material for: Managing Asthma Well and Sustainably – Patient Perspectives Explored
Source: Health Expect. 2026 Jul 3;29(4):e70751. doi: 10.1111/hex.70751 (PMC13332319; doi:10.1111/hex.70751)
Supplement: Supplementary file 3 — Supporting File 3: [file HEX-29-e70751-s002.docx]

# **S3. Inhaler interviews topic guide**

Thank you for taking the time to speak with me today. My name is Imogen and I am a research assistant at the University of Sydney. As we’ve already said, we are conducting interviews with people across Australia to explore your views and experiences with using respiratory inhalers.

The interview should take about 30 mins but do let me know if you need to leave earlier for any reason, that’s no problem.

*[Check have submitted eConsent form and agreed to recording]*

You indicated on your consent form that you are ok with me recording our conversation today, just so that I don’t miss any of your comments. Is that still ok?

I’ll also just remind you that you don’t have to respond to any questions that you don’t feel comfortable answering. Does that all sound ok? Let’s get started.

*[Check survey responses before interview] –* **fill in any blanks with participant if there is missing data.**

## **Warm up**

- Story of how the participant came to be using an inhaler/s

## **Inhaler use**

- If participant has not indicated type of inhaler on survey, begin by asking what type/s of inhaler/s they currently use *[can use NAC medications chart as visual guide if needed]*
- If participant has indicated type of inhaler:
  - From your survey responses, I can see that the type of inhaler/s that you currently use is/are [...............]. *[Clarify what they use for maintenance/prevention (if any) and what they use as emergency reliever.]*
  - Reasons for use
  - You may know that most inhalers are either pressurised metered dose or pMDIs or dry powder or DPIs.
    - Do you know the main difference between these?
    - Do you know if your inhaler is an MDI or a DPI?
  - *[If using a reliever]* Do you ever purchase any of your inhalers without a script from a doctor – at a pharmacy?
    - Reasons for over-the-counter purchasing?
- Pros and cons of currently used inhaler/s *[If participant uses more than one inhaler, make sure you keep these apart for each inhaler and/or ask which one specifically they are talking about.]:*
  - Explore any unwanted side effects, costs, convenience, ease of use, hygiene of inhaler, how quickly does relief inhaler provide relief.
  - What would you say is the one thing that you value most about your current inhaler(s)?
- Have you previously used any other inhalers or been offered other inhaler options? [*skip these questions if N/A*]
  - Why do you no longer use these inhalers?
  - (*if they know the difference):* Did you change from a DPI to an MDI (or other way round) or did the medication change or remain the same
  - How easy or difficult did you find changing inhalers (e.g. inhaler types or between medications). Reasons.
- If required, what would make you more (or less) willing to change your inhaler type to another?
  - E.g. ease of use, relief of symptoms, easier to access, cheaper, side effects (of current inhaler), better control of symptoms

## **Management**

You indicated that you have/have not got an asthma management plan

- If participant has a plan:
  - Do you mostly stick to the management plan recommended by your doctor or not?
  - Reasons
  - How often (if at all) do you see your doctor about your asthma or your treatment plan?
    - How often is the plan reviewed?
    - Is the plan ever modified, if yes, what is this based on (tests)?
- If participant does NOT have a plan:
  - Explore reasons re why no asthma management plan
  - Have you ever had a plan?

## **Attitudes**

- Do you feel that your condition is currently well managed?
  - What does ‘well-managed’ mean for you personally?

Prompts (if required):

- - - Frequency of using reliever/Ventolin /puffer
    - Frequency of exacerbations /asthma attacks/ problems breathing etc
    - Frequency of review/ doctor’s visits for asthma related issues
    - Frequency of sleep disturbance
    - Degree of impact on daily physical/ social activities
    - Degree of shortness of breath

## **Awareness and views on inappropriate medication use/overuse**

People commonly rely on Ventolin puffers for quick relief. But using this type of inhaler more than occasionally isn’t generally recommended *(in national guidelines).*

- Have you seen or heard this information before?
  - If yes, where/who from?
  - Prompt: Why do you think this information has not been shared with you?
  - What are your views on/how do you feel about this information?
  - If you currently use a Ventolin puffer, and you use it more than occasionally, would you be interested in speaking with your GP/specialist/pharmacist about this? Why/why not.

## **Awareness and views on carbon footprint of respiratory inhalers**

You might have heard that some types of inhalers are better for the environment than others because they have a smaller carbon footprint. [D*isplay figure with comparison between a DPI and MDI inhaler in terms of carbon and km travelled*] [Offer to clarify slide with P]

- Have you seen or heard this information before?
  - If yes, where/who from?
    - Do your doctor or pharmacist ever mention the environmental impacts of inhaler options for your condition?
    - Prompt: Why do you think this information has not been shared with you?
  - What are your views on /how do you feel about this information?
  - Would now knowing the environmental impact of inhalers impact your choice/ are you likely to discuss this with your doctor or pharmacist?

## **Wrap up**

- Is there anything else about respiratory inhalers or environmental issues in healthcare that we haven’t discussed yet that you think would be useful for the research team to know?

Thank you very much for your participation in this interview.

If this interview has raised any questions about your asthma care, please consult with your GP or specialist. Some good online resources you could look at and/or talk to your doctor about can be found by searching Asthma Australia or National Asthma Council. [*copy into chat for them if required*]: <https://asthma.org.au> <https://www.nationalasthma.org.au/>
