# Supplementary material for: Managing Asthma Well and Sustainably – Patient Perspectives Explored
Source: Health Expect. 2026 Jul 3;29(4):e70751. doi: 10.1111/hex.70751 (PMC13332319; doi:10.1111/hex.70751)

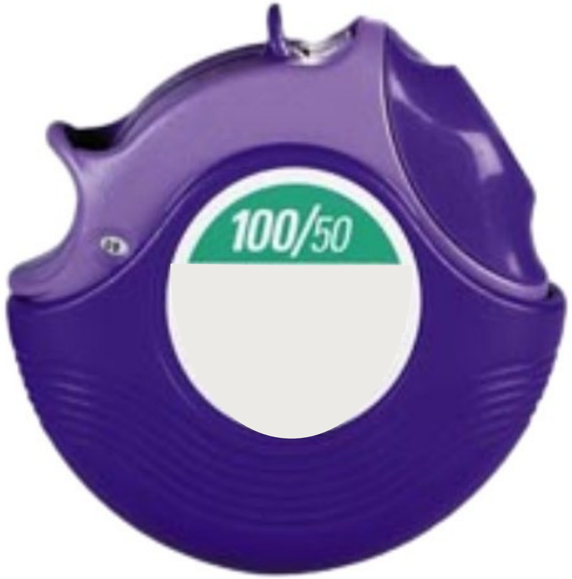

DPI vs MDI

What's the difference?

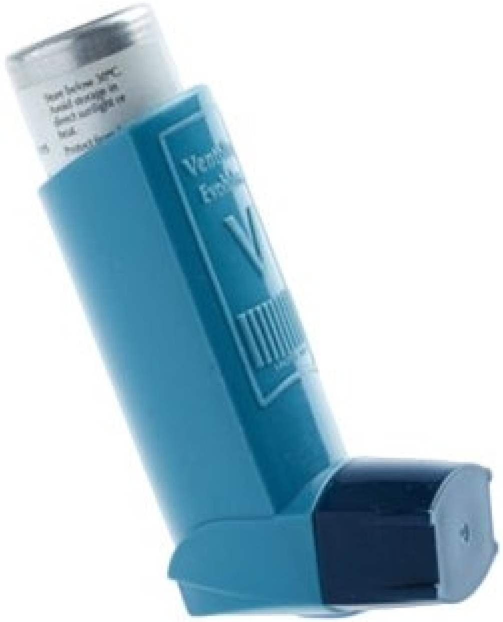

Dry Powder Inhaler

- No propellant
- < 1kg CO_2 equivalent emissions
- Equivalent to driving ~0.5km-4km

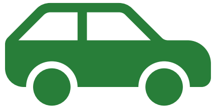

Metered Dose Inhaler

- Contains propellant
- up to 37kg CO_2 equivalent emissions
- Equivalent to driving ~ 40km-150km

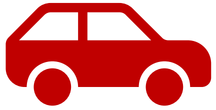

Supplement: Supplementary file 4 — Supporting File 4: [file HEX-29-e70751-s004.pdf]
